# Intimate Partner Violence and the Role of Child Maltreatment and Neighborhood Violence: A Retrospective Study of African American and US Caribbean Black Women

**DOI:** 10.3390/ijerph18052245

**Published:** 2021-02-24

**Authors:** Krim K. Lacey, Hira R. Shahid, Rohan D. Jeremiah

**Affiliations:** 1 Department of Sociology and African and African American Studies, University of Michigan-Dearborn, Dearborn, MI 48128, USA; 2 CASL, University of Michigan-Dearborn, Dearborn, MI 48128, USA; hrshahid04@gmail.com; 3 Human Development and Nursing Sciences, College of Nursing, University of Illinois-Chicago, Chicago, IL 60612, USA; rjerem@uic.edu

**Keywords:** intimate partner violence, child maltreatment, violent exposure, mental health

## Abstract

*Background*: Research suggests that intimate partner violence (IPV) is associated with childhood maltreatment and violence exposure within the neighborhood context. This study examined the role of child maltreatment and violence exposure on intimate partner violence, with the moderating effects of mental disorders (IPV) among US Black women. *Methods*: Data from the National Survey of American Life (NSAL), the largest and most complete sample on the mental health of US Blacks, and the first representative sample of Caribbean Blacks residing in the United States was used to address the study objectives. Descriptive statistics, chi-square test of independence, *t*-test, and logistic regression procedures were used to analyze the data. *Results*: Bivariate results indicate an association between child abuse and intimate partner victimization among US Black women. Witnessing violence as a child as well as neighborhood violence exposure was also related to IPV but shown to differ between African American and Caribbean Black women. Multivariate findings confirmed the influence of mental disorders and social conditions on US Black women’s risk for IPV. Moderating effects of child maltreatment and mental disorders in association with adult IPV were not found. *Conclusions*: The study addressed the short and long-term impact of child maltreatment and the contribution to the cycle of intimate violence among US Black women including African American and Caribbean Blacks. The study suggests the need for prevention and intervention efforts to improve structural conditions for at-risk populations and communities predisposed to violence and other negative outcomes. Possibilities for future research are also discussed.

## 1. Introduction

Approximately half (41.7%) of US Black women, including Caribbean women, currently comprising of one of the fastest-growing ethnic groups, have reported physical intimate victimization in their lifetime [1,2,3,4]. Studies show that early childhood exposure to violence within families and the neighborhood context are precursors to intimate partner violence as an adult [5]. Statistics indicate the rate of child abuse among African Americans in the United States is second highest only to American Indian and Alaskan Native [6]. While there is general knowledge surrounding the association between adult intimate partner violence and child abuse, less is known about the role of neighborhood violence context exposure on intimate victimization among US Black women [7]. To inform intervention and preventative practices, research geared to understanding the interconnectedness of violence at the individual and community levels is necessary due to high and rising levels of child abuse and reported cases of violence within the Black population [8,9,10,11].

Child abuse is one of many childhood adversities that can be a precursor for violence in adulthood [12]. Studies suggest the link between child maltreatment and adult intimate partner violence may be influenced by childhood experience and mental disorders [5,13]. However, there is a void in the literature on how social behavior and mental conditions might moderate the relationship between child maltreatment and adult intimate partner violence among US Black women and other understudied populations (i.e., Latinx, Native American, Asian American, Immigrant and Refugees). This study utilized population-based data to explore IPV in association with child maltreatment and neighborhood violence among US Black women with a specific focus on African American and Caribbean Blacks.

### 1.1. Background

Research has long found a connection between a history of exposure to violence and intimate victimization [5,11,14,15,16]. Studies largely suggest that experiencing or witnessing acts of aggression can influence perpetration or victimization [11,14,17,18]. Social learning theory posits that acts of violence are learned through imitation; and such acts internalized may influence our approach to addressing disputes in interpersonal relationships [19,20]. The framework evaluates behaviors that are normalized and rewarded while examining operant methods that provide explanations on how experiences with child abuse or exposure to violence may be linked to adult victimization [21]. For example, children who are exposed and/or socialized in violent-prone environments may be more accepting of certain behaviors, and therefore, are more likely to resort to such practices during their relationships in later life. The intergenerational transmission hypothesis further contends that violent behavior is learned through modeling and imitation, and such behavior is particularly acquired in the early life course during childhood and through observation of parents and peer relationships [22,23]. Moreover, violence within the home or against children is rooted in the subconscious and intergenerational cycle of violence that perpetuates from one generation to the next [24,25].

### 1.2. Child Abuse and Intimate Partner Violence

Studies have demonstrated that there is a co-occurrence of child abuse and adult victimization [5,11,14,15,18,26]. Notably, harsh physical treatment and disciplinary measures in childhood have been found to increase the association of violence in adulthood [8,21]. For example, children growing up in violent homes are at risk of becoming victims of IPV [27]. Women in particular, who were raised in violent households, are at greater risk for suffering and becoming victims of intimate partner violence [26]. Although there is a general knowledge about the potential connection between child abuse and intimate partner violence, the understanding among ethnic groups where physical punishment is a method of disciplinary practice used by a parent or caretaker in rearing children, remains limited [28]. Nonetheless, there is some evidence that suggests childhood victimization increases the risk for physical, psychological, and sexual victimization and perpetration into adulthood among Caribbeans [29]. In recent years, more emphasis has been applied to understand the nuance of this problem from a more intersectional perspective [30,31]. However, more inquiries are needed to understand the association between child abuse and intimate partner violence among U.S. Black women, who are more vulnerable to victimization compared to other populations [1]. While considerable progress has been made to understand these issues among Black Americans, there are still considerable gaps that delineate the experiences of Caribbean Americans, a growing sub-population, that has its own experience with violence.

### 1.3. Childhood Exposure to Violence and Intimate Partner Violence

Along with direct acts of child abuse research further recognizes that children’s exposure to violence increases the risk for adult perpetration and victimization, as well [5,13,18,32]. Particularly, children who bear witness to, or are exposed to family violence, were found to be at increased risk of battering later in life [5,13,32]. Research has established that early exposure to family violence results in males being 3 to 10 times more prone to partner violence than males without exposure to violence [26]. The connection between children’s exposure to violence and adult intimate perpetration and victimization has also been noted by various international studies. For example, women in Jamaica who had witnessed parental or family violence were found more likely to be physically abused by an intimate partner [33]. Gage earlier found that compared to Haitian women who had not observed their father beating their mothers, those who had such experiences reported significantly higher rates of emotional and sexual violence [34]. In Grenada, Jeremiah et. al. explored how the failure to address adverse childhood experiences—such as witnessing abuse among their parents continued to affect adult women that were associated with domestic violence [35]. Despite these findings, we still lack an understanding of the role of cultural norms in the relationship between children’s exposure to violence and the risk for adulthood victimization.

### 1.4. Neighborhood Violence Exposure and Intimate Partner Violence

While the literature is relatively new in providing an understanding of the effects of neighborhood violence on intimate partner violence, the association has been mixed [17,18,36]. Reed and colleagues (2009) established that neighborhood violence in addition to perception about intimate partner violence is associated with increased perpetration of IPV among urban African American men [10]. A systematic review further links neighborhood environment and disadvantage with physical and sexual IPV while noting the influence of socio-economic factors (i.e., poverty, unemployment, income, education) [17]. This was supported by an earlier study that linked neighborhood disadvantage to IPV [37]. Conversely, little variation was found in the likelihood of male IPV concerning neighborhood crime in other studies, even though there was an increased likelihood of IPV experiences among women whose partners were involved in male-to-male violence [38]. Raghavan and colleagues additionally found that living in a neighborhood with high levels of social disorder and substance use increased women’s exposure to community violence and subsequent IPV in adulthood [7].

### 1.5. Mediating and Moderating Effects of Child Maltreatment and Intimate Partner Violence

Research suggests that the relationship between later intimate partner violence and child maltreatment including witnessing violence and child abuse, is not always linear and may be influenced by childhood adversities and mental conditions [39,40]. For some children or adolescents, the possibility of developing emotional and psychological problems in response to painful experiences with exposure to violence is not out of the ordinary during this critical stage of development. Studies have found experiences with maltreatment are accompanied by external and antisocial behaviors [5,18,40]. The association between child maltreatment and adult victimization may also be reflective of hostile behaviors often developed by abused children [18,40], particularly if the childhood trauma goes unacknowledged or untreated. Such hostile behavioral patterns, which may be a part of their coping strategy, are poor impulse control that may be present among perpetrators of violence [26].

There is evidence that child maltreatment might further be linked to the use and abuse of alcohol and other substances. Early substance abuse among children and adolescents is common, and often related to aggressive behavior that can continue into adult life [41,42]. Research additionally suggests personality disorders may have some influence on partner violence [40]. Ehresaft et al. found that personality disorder partially mediated the relationship between childhood family violence and adult partner violence [43]. Likewise, a prospective longitudinal study found that early behavior problems were associated with partner violence in adulthood [42]. Conduct disorder, in particular, was found to mediate the relationship between child abuse and partner violence [5,41]. Furthermore, conduct disordered behavior in early childhood and adolescence has been linked to IPV perpetration in later adulthood [44]. Irrespective of previous studies, potential moderators or mediators of intimate partner violence such as substance abuse, anti-social personality disorder, conduct disorder, and oppositional defiant disorder have yet to be fully explored in the relationship among US Black women using national data.

### 1.6. Goals and Summary of Hypothesis

Using a nationally representative sample, the present study sought to add to the body of knowledge by exploring the association between violence exposure and intimate partner violence among US Blacks with a focus on African American and Caribbean Black women. The specific aims of the study were to: (a) examine the relationship between child maltreatment (child abuse or witnessing violence as a child) and adult severe physical intimate partner violence (SPIPV); (b) address the relationship between exposure to neighborhood violence and intimate partner violence; (c) and to evaluate the moderating effects of substance abuse disorder, conduct disorder, anti-social personality disorder and oppositional defiant disorder in association with child abuse and later interpersonal violence. As with previous studies, we expected to find an association between severe intimate partner violence and both child maltreatment and exposure to neighborhood violence. We also expected that the relationship between child maltreatment and intimate partner violence would be moderated by mental disorders.

## 2. Materials and Methods

Data from the National Survey of American Life (NSAL), conducted over a three-year period between 2001–2003, were used to address the research aims. The NSAL to date is the most comprehensive study conducted on the mental and physical health of adult US Blacks, and the first nationally representative study of Caribbean Blacks residing in the United States (see Jackson et al. [45]). Multistage probability sampling methods were used to collect the data. Face-to-face interviewing was the primary method of data collection, with a smaller percentage (14%) collected by phone. In total, the sample consisted of 6082 participants: 3570 African American; 1621 Caribbean Black; and 890 non-Hispanic White respondents. African Americans were characterized as those with African ancestry but without Caribbean roots. Caribbean Blacks were those respondents of African descent who were either (a) of West Indian descent, (b) from a Caribbean-area country, or (c) had parents or grandparents who were born in a Caribbean area country [46]. Prior to the data collection process, informed consent was obtained from participants. Interviews on average were 2 h and 20 min in length. The response rate for the entire sample was 72.3 percent. Respondents received an honorarium of $50 for their participation in the study. For this study, approximately 3277 women of African descent were the focus of analysis. Data collection for the NSAL was approved by the University of Michigan’s Institutional Review Board.

### 2.1. Predictor Measures

#### 2.1.1. Control Variables 

The control variables included age (in years), marital status, employment status, educational level, and poverty. Marital status was separated into married, partnered, separated or divorced, widowed, or never married. Employment status was divided into employed, unemployed, and not in the labor force. Educational level included less than high school, high school graduates, some college, and college-educated. Poverty status is an income-to-poverty ratio consisting of the participants’ household income divided by the 2001 US Census poverty threshold for the number of adults and children living in that household. Ratios below 1.00 indicate that the income for the participants’ household is below the official poverty threshold, while a ratio of 1.00 or greater indicates income above the poverty level. For example, a ratio of 1.25 indicates that income was 25 percent above the appropriate poverty threshold [47]. Two ethnic groups were examined: African Americans and Caribbean Blacks. Noted earlier, African Americans were persons who self-identified as Black but did not report Caribbean ancestry. By contrast, US Caribbean Blacks were persons who were descendants or had Caribbean roots [46]. US Blacks were inclusive of both ethnic groups.

#### 2.1.2. Child Maltreatment 

Child maltreatment is inclusive of two measures: child abuse and witnessing violence as a child. Child abuse was determined by the question, “As a child, were you ever badly beaten up by your parent or the people that raised you?” Response options were “yes” or “no.” For witnessing violence, respondents were asked, “When you were a child, did you ever witness serious physical fights at home, like when your father beat up your mother (yes/no)?” These measured were combined for multivariate analysis.

#### 2.1.3. Neighborhood Violence

Various markers of neighborhood violence exposure were used in the study. First, experiences with neighborhood crime were operationalized with the question, “How often are there problems with muggings, burglaries, assaults, or anything like that in your neighborhood?” Measured on Likert scale response options include: very often, fairly often, not too often, hardly ever, and never. The variable was recoded to reflect ever/often vs. never for bivariate analysis. Second, to address experiences with atrocities, respondents were asked, “Did you ever see atrocities or carnage such as mutilated bodies or mass killings (yes/no)?” Third, seen someone badly injured, was determined by the question, “Did you ever see someone being badly injured or killed, or unexpectedly see a dead body (yes/no)?”

#### 2.1.4. Moderators

A modified version of the World Health Organization Composite International Diagnostic Interview (WHO CIDI) defined by the Statistical Manual of Mental Disorders, Fourth Edition (DSM IV) was used to obtain information on respondents that met criteria for substance abuse disorder, antisocial personality disorder, conduct disorder, and oppositional defiant disorder (yes/no) [48]. Substance abuse disorder refers to the presence of either alcohol or drugs, or both. In addition to alcohol, the substances included are cocaine, tranquilizers, stimulants, pain killers, other prescription drugs, such as heroin, opium, glue, LSD, peyote, or any other controlled substance. The criteria for substance abuse do not include drug-related consequences of tolerance, withdrawal, or a pattern of compulsive use, and instead include only the harmful consequences of repeated use. Antisocial personality disorder (APD) is a pervasive pattern of disregard for, and violation of, the rights of others that begins in childhood or early adolescence and continues into adulthood. The pattern has also been referred to as psychopathy, or sociopathy. Because deceit and manipulation are central features of APD, it may be especially helpful to integrate information acquired from collateral sources. For the diagnosis to be given, the individual must be at least 18 years of age and must have had a history of some symptoms of conduct disorder before age 15. Conduct disorder (CD) involves a repetitive and persistent pattern of behavior in which the basic rights of others or major age-appropriate societal norms or rules are violated. The specific characteristics of conduct disorder fall into one of four categories: aggression to people or animals, destruction of property, deceitfulness or theft, or serious violation of rules. The symptoms of CD include three or more of the following: deceitfulness, impulsivity, irritability or aggressiveness, reckless disregard for safety, irresponsibility, or lack of remorse. Oppositional defiant disorder (ODD) is a recurrent pattern of negativistic, defiant, disobedient, or hostile behavior toward authority figures that persists for at least 6 months, and is characterized by the frequent occurrence of at least four of the following: losing temper, arguing with adults, actively defying/refusing to comply with the rules of adults, deliberately doing things that will annoy other people, blaming others for his or her own mistakes or misbehavior, being touchy or easily annoyed, being angry and resentful, or being spiteful or vindictive. These behaviors occur more frequently than is typically observed in individuals of comparable age and must lead to significant impairment in functioning.

### 2.2. Outcome Measure

#### Intimate Partner Violence

IPV was operationalized with the question: “Have you ever been badly beaten up by a spouse or romantic partner?” Response options were “yes” and “no.” We assessed this single measure’s validity by comparing it to the National Comorbidity Study Replication (NCS-R) dichotomously defined Conflict Tactic Scale within the Collaborative Psychiatric Epidemiology Surveys (CPES) [49,50]. Two tests were conducted to assess the measure’s validity. The probability of agreement (OR = 4.5, *p* < 0.001) [51,52,53], and area under the curve (AUC > 0.6) showed the item to have a fair association across estimates [54]. Five hundred and five (*n* = 505) Black women in the sample reported severe physical intimate partner violence.

### 2.3. Analytic Strategy

Descriptive statistics and bivariate (chi-square test, *t*-test) analytic procedures were employed to provide information on the sample distribution and SPIPV in associations with child maltreatment by a parent or caretaker, and neighborhood violent exposure within cohorts (e.g., US Black women, African American, Caribbean Black). Simultaneous multivariate logistic regression analysis was conducted to address the association between child maltreatment and adult intimate partner violence controlling for other factors. Within the analysis, moderating effects were assessed by including interaction terms for child maltreatment and mental disorders (e.g., child maltreatment X substance disorder). For these procedures, adjustments were made for complex sample design. Due to the underlying complex sample design, standard errors were corrected for weighting, clustering, and stratification. Adjustments were made for complex sample design and differential non-response. Stata 15.1 analytical software was used to produce statistical results. Significance was set at the 0.05 alpha level. Diagnostic test revealed an acceptable variance inflation factor (VIF), limiting collinearity concerns for the multivariate model.

### 2.4. Sample Characteristics

The average age of women within the sample was forty-three years (*m* = 42.5) old (see Table 1). A third (32%) of respondents never married. The socio-economic status of participants within the sample was different. Specifically, thirty-six percent of participants had a high school diploma. Almost two-thirds (63.7%) of respondents were employed. Meanwhile, nearly three-quarters (71.6%) of women lived at or above the federal poverty level. Finally, the majority of women in the sample were African American (93.8%).

## 3. Results

### 3.1. Analysis Examining the Association of Adult IPV, Child Maltreatment and Neighborhood Violent Exposure Variables among US Black Women

Illustrated by Table 2 on US Black women in general, the rate at which they experienced child abuse was more than three times the percentage for victims of severe physical intimate partner violence (SPIPV) than non-victims (13.5% vs. 3.9%, *p* < 0.001). For women who witnessed violence in the household as a child, the percentage of SPIPV was almost two-fold that of non-victims (36.3% vs. 17.6%, *p* < 0.001). There were significantly higher percentages of SPIPV victims compared to non-victims (36.3% vs. 21.4%, *p* < 0.001) among respondents who had seen someone injured.

Among African American women, similar results were found as those previously noted (see Table 3). The proportion of severe physical intimate partner violence exceeded that of non-victims by three-fold (13.1% vs. 3.9%, *p* < 0.001) for those who were the victim of child abuse. For respondents that witnessed violence in the household, the rates were significantly higher (36.7% vs. 17.9%, *p* < 0.001) among those that experienced SPIPV than non-victims of IPV. The same was true for women who had seen someone injured; the percentage was significantly higher (36.0% vs. 20.9%, *p* < 0.001) for those who experienced severe intimate partner violence than non-victims.

The proportion of severe physical intimate partner victims significantly exceeded that of non-victims (22.2% vs. 3.3%, *p* < 0.001) for Caribbean Black women who reported child abuse (see Table 4). Among those women exposed to neighborhood violence, there was a higher percentage of SPIPV victims than non-victims (94.3% vs. 82.2%, *p* < 0.05). Although marginally significant, the percentage at which those who witnessed violence in the household were twice that for IPV victims than non-victims of SPIPV (27.8% vs. 12.9%, *p* = 0.064).

### 3.2. Multivariate Analysis Examining Associations and Moderating Factors of Intimate Partner Violence among US Black Women

Multivariate results show that the odds (AOR = 4.07, *p* < 0.05 CI 1.11, 14.92, *p* < 0.05) for severe physical intimate partner violence significantly increased among women who reported child maltreatment (see Table 5). In the absence of child maltreatment, however, there were other influences of severe physical intimate partner violence. First, the odds (AOR = 2.35, CI = 1.33, 4.16, *p* < 0.01) for SPIPV increased among women who met criteria for conduct disorder. Furthermore, anti-social personality disorder both increased the possibility (AOR = 4.87, CI = 2.28, 10.41, *p* < 0.001) and probability (AOR = 1.74, CI = 0.942, 3.22, *p* = 0.076; CI) of SPIPV. Moderating effects were not found between child maltreatment and severe intimate partner violence.

The study results also indicate an association between socio-demographic factors and severe physical intimate partner violence. Notably, the odds (AOR = 1.06, CI = 1.03, 1.08, *p* < 0.001) for severe intimate partner violence increased with age. Compared with married respondents, those separated or divorced were at increased odds (AOR = 2.52, CI = 1.25–5.06, *p* < 0.01) for SPIPV. Finally, the odds (AOR = 1.77, CI = 1.13, 2.77 *p* < 0.01) for SPIPV increased among participants living at or below poverty almost by two-fold compared with those living above the poverty threshold. Collectively the independent variables in the model explained 14 percent of the variance in severe intimate partner violence.

## 4. Discussion

The results of the study provide theoretical support for social learning theory and the intergenerational transmission model. By and large, the study indicates that child abuse is linked to victimization in adulthood among US Black women, as with other populations [5,33,55]. This was evident in bivariate analysis across cohorts. While there was an association between exposure to violence and severe intimate partner violence, our study revealed that these experiences differ by ethnic groups. For Caribbean Black women, exposure to neighborhood violence was associated with adult victimization. Meanwhile for African Americans, witnessing violence and seeing someone injured was related to severe intimate partner violence. The findings and differences found between ethnic groups are difficult to explain. Quite possibly, this might be influenced by the cultural differences between Caribbean Blacks and African Americans in terms of how violence is defined and interpreted. However, the findings may point to the commonality of social, economic, and environmental conditions facing Blacks in the United States, including sources of stress, which might expose women to subsequent victimization. As evident in this study, racial and ethnic minorities including Caribbean blacks and African Americans face issues of poverty rate which might confine them to neighborhoods with higher criminal activities and violence, less economic resources and opportunities. There is also evidence that immigrant groups may more so face these challenges due to high rates of poverty after arrival and the absence of generational capital that has been accumulated by US Blacks [56].

Even though there was confirmation regarding the association between child maltreatment and severe intimate victimization in multivariate analysis, our study, in general, did not find any support concerning the moderating effects of mental health disorders. Nonetheless, we did find that independent of child maltreatment, the risk for adult intimate partner violence increased among US Black women who met criteria for conduct and anti-personality disorders. Although these results were either partially or fully supported by previous studies [40,57,58,59], the mechanism by which these factors influence intimate partner violence among US Black women is less clear.

The study results further showed that separated or divorced Black women were at an increased risk for intimate victimization. This could reflect the escalation of violence after the women leave or attempt to leave the relationship [60]. It should also be noted that some divorced or separated women may still be in abusive relationships, even though their relationship status had changed. Additionally, this research found that violence among women within this population increased with age. This finding contradicts other research trends that find that exposure to violence generally reduces with age [61]. Finally, women living in poverty were found to be at increased risk for intimate partner violence, which is consistent with previous research [5,62,63,64,65,66]. Known to many, poverty contributes to stress and increases the possibility of violent explosive encounters in relationships [67].

### 4.1. Limitations of the Study

We acknowledge that this study has a few limitations. First, cross-sectional data were used for this study, limiting causal inferences about the relationship between intimate partner violence and both child maltreatment and neighborhood violent exposure. As such, it is difficult to determine the temporal ordering of the relationship. Studies using longitudinal data are necessary for clarifying these relationships. Second, the study was retrospective and may be subject to recall bias, especially for those who have experienced victimization in early life. Therefore, memory lapses could cause participants to attribute certain conditions to other traumatic experiences. Third, the data used for this study is over a decade old and may not reflect current events, though the relationship is not likely to change over this period [68]. The data used for the study to our knowledge is the only available national data that allowed for the examination of the study goals, particularly in respect to ethnic groups within the US Black population. Furthermore, a single binary measure was used to address severe physical intimate partner violence. Even so, such a measure has been used in studies of this nature before [54,64]. Moreover, a comparison of the NSAL IPV indicator with the CTS found a fair agreement with the measure [54]. The measure used for this study also allowed for examining the relationship between child maltreatment, violence exposure, and adult victimization among black women. Additionally, only physical intimate partner violence was examined in this study. Other forms (e.g., psychological/emotional/verbal) of abuse were not evaluated due to data limitations. Finally, sample size issues prevented us from independently examining the moderating effects of child abuse and witnessing violence as a child on the mental disorders of women in this study.

### 4.2. Benefits of the Study

Despite the limitations, this study sheds light on the issue of child maltreatment as an important factor in the trajectory of victimized Black women, which has been lacking empirically using data at the national level. The study also addressed the contribution of other disorders on intimate partner violence. This research further provides insights into the significance of the potential role of structural and environmental conditions that are prominent in the lives of US Blacks which might influence victimization. Finally, this research highlights that while similar in some regards, there are differences that exist ethnically and culturally regarding the association of child maltreatment and neighborhood conditions in relation to Black women’s experience with intimate partner violence.

## 5. Conclusions

The study has implications for prevention and intervention strategies for IPV. More notably, the findings reinforce both the short- and long-term outcomes of child abuse and witnessing violence as a child. Along with the immediate traumatic effects on the health and well-being of children, child maltreatment can contribute to a cycle of violence that has been known to influence intimate partner victimization or perpetration, placing US Black women at risk for poor outcomes. While some ethnic groups continue to endorse physical punishment, it is becoming more apparent that this method of discipline can contribute to a larger problem in later life. Therefore, other forms of non-violent disciplinary measures should be considered at the earliest stage of the life course. Along with child abuse, the study suggests possible exposure to violence resulting from poor social, economic, and environmental conditions circumstances may serve as a precursor for future violence. Hence, there is a need for preventative measures particularly in impoverished areas where individuals are likely to face these realities in their homes and neighborhoods. Likewise, primary, secondary and tertiary prevention of childhood exposure to violence (group and one on one counseling, addressing defiant behavior, engaging in restorative justice, etc.) can be used as a deterrence for adult IPV victimization and perpetration. Finally, additional studies are necessary to better understand the general and mediating effects of conduct and antisocial disorders and their association with intimate partner violence within minority populations.

## Figures and Tables

**Table 1:** Sample Characteristics (N = 3277).

Variable	Total Sample (%)	Ever Report IPV	No IPV Report	F-Test Statistics
Age (mean)	42.5	42.7	42.0	0.46
Relationship Status				10.86 ***
Married	27.4	20.6	28.7	
Partnered	8.4	10.0	8.2	
Separated or Divorced	20.3	33.6	17.7	
Widowed	11.5	8.9	11.7	
Never Married	32.4	26.9	33.8	
Education				5.74 *
Less than High School	24.8	34.1	23.1	
High School Diploma	36.0	31.1	36.3	
Some College	24.8	24.0	25.2	
College	14.4	10.8	15.4	
Employment Status				6.82 *
Employed	63.7	58.8	65.0	
Unemployed	11.1	16.1	10.2	
Not in the Labor Force	25.2	25.1	24.7	
Poverty				14.95 **
At or Above	71.6	62.2	73.4	
Below	28.4	37.8	26.6	
Ethnicity				
African American	93.8	93.3	95.7	4.83 *
Caribbean Black	6.2	6.7	4.3	

Note. Statistics are weighted; * *p* < 0.05 ** *p* < 0.01 *** *p* < 0.001.

**Table 2:** Bivariate Analysis Examining Child Maltreatment, Violence Exposure on SPIPV and non-SPIPV US Black Women.

Variables	Total	Ever Report IPV	No IPV Report	Unadjusted OR	*p*-Value
Child Abuse					
No	94.4	86.5	96.1		
Yes	5.6	13.5	3.9	3.85 ***	0.000
Neighborhood Crime					
Never	24.0	20.4	24.1		
Very/often	76.0	79.6	75.9	1.24	1.000
Witnessing Violence					
No	79.1	63.7	82.4		
Yes	20.9	36.3	17.6	2.66 ***	0.000
Exposure to Atrocity					
No	98.2	98.0	98.2		
Yes	1.8	2.0	1.8	1.13	1.000
Seen Someone Injured					
No	75.9	63.7	78.6		
Yes	24.1	36.3	21.4	2.09 ***	0.000

Note. There were 505 US Black women in the sample who reported severe physical intimate partner violence. *** *p* < 0.001.

**Table 3:** Bivariate Analysis Examining Child Maltreatment, Violence Exposure on SPIPV and non-SPIPV African American Women.

Variables	Total	Ever Report IPV	No IPV Report	Unadjusted OR	*p*-Value
Child Abuse					
No	94.4	86.9	96.1		
Yes	5.6	13.1	3.9	3.67 ***	0.000
Neighborhood Crime					
Never	24.4	21.1	24.6		
Very/often	75.6	78.9	75.4	1.22	1.000
Witnessing Violence					
No	78.7	63.3	82.1		
Yes	21.3	36.7	17.9	2.65 ***	0.000
Exposure to Atrocity					
No	98.2	97.9	98.3		
Yes	1.8	2.1	1.7	1.23	1.000
Seen Someone Injured					
No	76.3	64.0	79.1		
Yes	23.7	36.0	20.9	2.12 ***	0.000

Note. There were 392 African American women in the sample that reported severe physical intimate partner violence. *** *p* < 0.001.

**Table 4:** Bivariate Analysis Examining Child Maltreatment, Violence Exposure on SPIPV and non-SPIPV US Caribbean Black Women.

Variables	Total	Ever Report IPV	No IPV Report	Unadjusted OR	*p*-Value
Child Abuse					
No	94.4	77.6	96.7		
Yes	5.6	22.2	3.3	8.43 **	0.001
Neighborhood Crime					
Never	17.1	5.7	17.8		
Very/often	82.9	94.3	82.2	3.57 *	0.033
Witnessing Violence					
No	85.3	72.2	87.1		
Yes	14.7	27.8	12.9	2.59	0.064
Exposure to Atrocity					
No	97.7	100.0	97.4		
Yes	2.3	0.0	2.6	1.00	1.000
Seen Someone Injured					
No	69.8	56.4	71.6		
Yes	30.2	43.6	28.4	1.95	1.000

Note. There were 113 Caribbean Black women in the sample that reported severe physical intimate partner violence. * *p* < 0.05 ** *p* < 0.01.

**Table 5:** Multivariate Analysis Predicting Adult Severe Physical Intimate Partner Violence.

Variable	Odds Ratio	SE	*p* Value	95% CI
Age	1.06	0.014	0.000 ***	1.03–1.08
Education Level				
Less Than HS	1			
High School Graduate	0.73	0.162	0.159	0.465–1.14
Some College	0.83	0.206	0.461	0.507–1.37
College	0.59	0.204	0.135	0.298–1.18
Marital Status				
Married	1			
Partnered	0.98	0.361	0.950	0.466–2.05
Separated-Divorced	2.51	0.878	0.010 **	1.25–5.06
Widowed	3.60	4.21	0.277	0.347–37.37
Never Married	1.07	0.276	0.789	0.640–1.80
Race/Ethnicity				
African American	1			
Caribbean Black	0.88	0.246	0.644	0.501–1.54
Poverty Level				
Above	1			
Below	1.77	0.396	0.014 *	1.23–2.77
Employment Status				
Employed	1			
Not employed	1.14	0.162	0.159	0.465–1.14
Not in Labor Force	1.24	0.310	0.385	0.298–1.18
Child Maltreatment				
No	1			
Yes	4.07	2.64	0.035 *	1.11–14.92
Crime Problem in Neighborhood	1.02	0.091	0.854	0.849–1.22
Atrocities				
No	1			
Yes	0.68	0.413	0.529	0.201–2.30
Injury				
No	1			
Yes	1.36	0.268	0.126	0.915–2.02
Anti-Social Disorder				
No	1			
Possible	4.87	1.85	0.000 **	2.28–10.41
Probable	1.74	0.535	0.076	0.942–3.22
Child Maltreatment X Anti-Social Disorder				
No	1			
Possible	0.37	0.295	0.218	0.077–1.81
Probable	1.01	0.655	0.987	0.276–3.70
Substance Abuse				
No	1			
Yes	1.63	0.521	0.130	0.861–3.09
Child Maltreatment X Substance Abuse				
No	1			
Yes	1.27	0.667	0.657	0.440–3.63
Oppositional Defiant Disorder (ODD)				
No	1			
Yes	0.93	0.316	0.834	0.472–1.84
Child Maltreatment X Oppositional Defiant Disorder				
No	1			
Yes	0.39	0.259	0.161	0.101–1.48
Conduct Disorder (CD)				
No	1			
Yes	2.35	0.669	0.004 **	1.33–4.16
Child Maltreatment X Conduct Disorder				
No	1			
Yes	0.526	0.246	0.175	0.207–1.34

Note. * *p* < 0.05 ** *p* < 0.01 *** *p* < 0.001.

## Data Availability

The public use NSAL data is archived at the University of Michigan-ICPSR.
